# Haematometrocolpos Mimicking Appendicitis in an Adolescent Female: A Case Report

**DOI:** 10.7759/cureus.82726

**Published:** 2025-04-21

**Authors:** Wilhelm Hansen, Mohammed Fayaz Kimmie

**Affiliations:** 1 General Surgery, University of Witwatersrand, Johannesburg, ZAF; 2 General Surgery, Robert Mangaliso Sobukwe Hospital, Kimberley, ZAF

**Keywords:** appendicitis, diagnostic dilemma, general surgery, gynaecology, haematometrocolpos

## Abstract

This report presents the case of a 15-year-old female patient who exhibited symptoms of abdominal distension, constipation, and cyclical lower abdominal pain. Subsequent evaluation led to a diagnosis of haematometrocolpos as a consequence of an imperforate hymen. Imaging studies confirmed substantial blood accumulation within the vaginal and uterine cavities, necessitating surgical intervention. The patient was subsequently referred to the gynaecological department for further management. This case underscores the significance of recognising haematometrocolpos as a differential diagnosis in adolescent females presenting with primary amenorrhoea and abdominal pain. Given the overlap in presentations between surgical and gynaecological conditions, timely surgical consultation and imaging are imperative to prevent unnecessary interventions, while prompt diagnosis and appropriate surgical management are crucial to avert complications and preserve reproductive function. Furthermore, this case underscores the necessity for comprehensive clinical examination and imaging in patients with obstructive Müllerian anomalies, highlighting the vital role of the general surgeon in the initial assessment and the exclusion of acute surgical emergencies.

## Introduction

Acute abdominal pain in adolescent females frequently constitutes a common clinical presentation characterised by a wide differential diagnosis. This often necessitates urgent evaluation to rule out surgical emergencies, such as appendicitis [1-3]. Nevertheless, in post-pubertal patients, gynaecological conditions require consideration, particularly when there is a documented history of primary amenorrhoea or cyclical pelvic pain. Haematometrocolpos, which arises from obstructed menstrual outflow, is a rare yet significant aetiology of acute abdominal pain within this demographic. The condition is commonly attributed to an imperforate hymen, but may also stem from a transverse vaginal septum or other Müllerian anomalies [4,5]. Patients may exhibit symptoms that mimic appendicitis; however, atypical presentations can arise due to the mass effect on adjacent structures, occasionally leading to urinary or gastrointestinal complaints. Early recognition of haematometrocolpus is essential to avoid misdiagnosis and ensure appropriate management, particularly when patients first present to general surgeons. Prompt identification is essential to avert complications such as retrograde menstruation, endometriosis, or secondary infection. This case underscores the importance of a broad differential in acute abdominal pain, its differentiation from more prevalent surgical conditions, and the pivotal role of the general surgeon in its initial assessment and management.

## Case presentation

A 15-year-old female patient with no known comorbidities presented with a two-week history of right iliac fossa (RIF) pain. The pain was noted to be non-radiating and was accompanied by symptoms of nausea, vomiting and decreased appetite. There were no reported urinary symptoms, but the patient experienced constipation. The patient's mother reported that she had not yet started menstruating. Initially, she sought assistance at a local clinic and was subsequently admitted to a community hospital, where she was administered analgesics and antibiotics (no blood results available). Despite these treatments, her clinical condition did not exhibit any sign of improvement, and she was referred to general surgery at a regional hospital for further management.

Upon admission, her vital signs were recorded as follows: blood pressure 132/83 mmHg, heart rate 125 beats per minute, respiratory rate (RR) 20 breaths per minute, oxygen saturation 98% on room air and temperature 36.7°C. Urinalysis indicated 1+ protein and 2+ ketones, alongside a negative urine beta-human chorionic gonadotropin (β-hCG) test.

During the general examination, findings were largely unremarkable. However, an abdominal examination revealed tenderness in the RIF with notable fullness, suggestive of a palpable mass upon palpation and rebound tenderness. Other systemic examinations were also unremarkable. Blood tests indicated mild leukocytosis, mild anaemia, thrombocytosis, mildly raised amylase, normal C-reactive protein, and no significant electrolyte abnormalities. Venous blood gas and co-oximetry results were within normal ranges. Detailed results are provided below (Tables 1, 2).

**Table 1:** Blood results

Day of Hospital Stay	Specimen Type	Result	Reference Range
1	Blood	Sodium: 134	135 - 145 mmol/L
		Potassium: 4.1	3.5 - 5.0 mmol/L
		Chloride: 102	98 - 107 mmol/L
		Bicarbonate: 19	22 - 28 mmol/L
		Urea: 5.1	2.5 - 7.8 mmol/L
		Creatinine: 58	45 - 90 µmol/L
		Amylase: 124	30 - 110 U/L
		Lipase: 26	13 - 60 U/L
		C-reactive protein: 1	<10 mg/L
		White cell count: 11.34	4.0 - 11.0 × 10⁹/L
		Haemoglobin: 11.7	12.0 - 16.0 g/dL
		Platelets: 601	150 - 400 × 10⁹/L

**Table 2:** Venous blood gas

Day of Hospital Stay	Specimen Type	Result	Reference Range
1	Blood	pH: 7.36	7.35 - 7.45
		pCO₂: 36	35 - 45 mmHg
		pO₂: 42	30 - 50 mmHg (venous)
		Sodium: 136	135 - 145 mmol/L
		Potassium: 4.0	3.5 - 5.0 mmol/L
		Chloride: 104	98 - 107 mmol/L
		Calcium: 1.23	1.15 - 1.35 mmol/L
		Hematocrit: 36	36 - 46%
		Glucose: 6.0	3.9 - 6.1 mmol/L
		Lactate: 1.8	0.5 - 2.2 mmol/L
		Total haemoglobin: 11.5	12.0 - 16.0 g/dL
		Oxygen saturation: 56.8%	60 - 80% (venous)
		Bicarbonate: 20.3	22 - 28 mmol/L
		Base excess: -5.1	-2 to +2 mmol/L
		Anion gap: 16	8 - 16 mmol/L

A formal ultrasound was booked for the patient. Ultrasound findings indicated extensive, mass-like thickening of the caecal wall, accompanied by a significant defect in the wall. A large, complex pelvic collection was observed, exerting compressive effects on the bladder. These findings strongly suggested an appendiceal perforation. Concern arose due to the insufficient detail in the gynaecological findings on ultrasound, prompting the treating team to order a computed tomography (CT) scan for a more thorough assessment.

**Figure 1:**
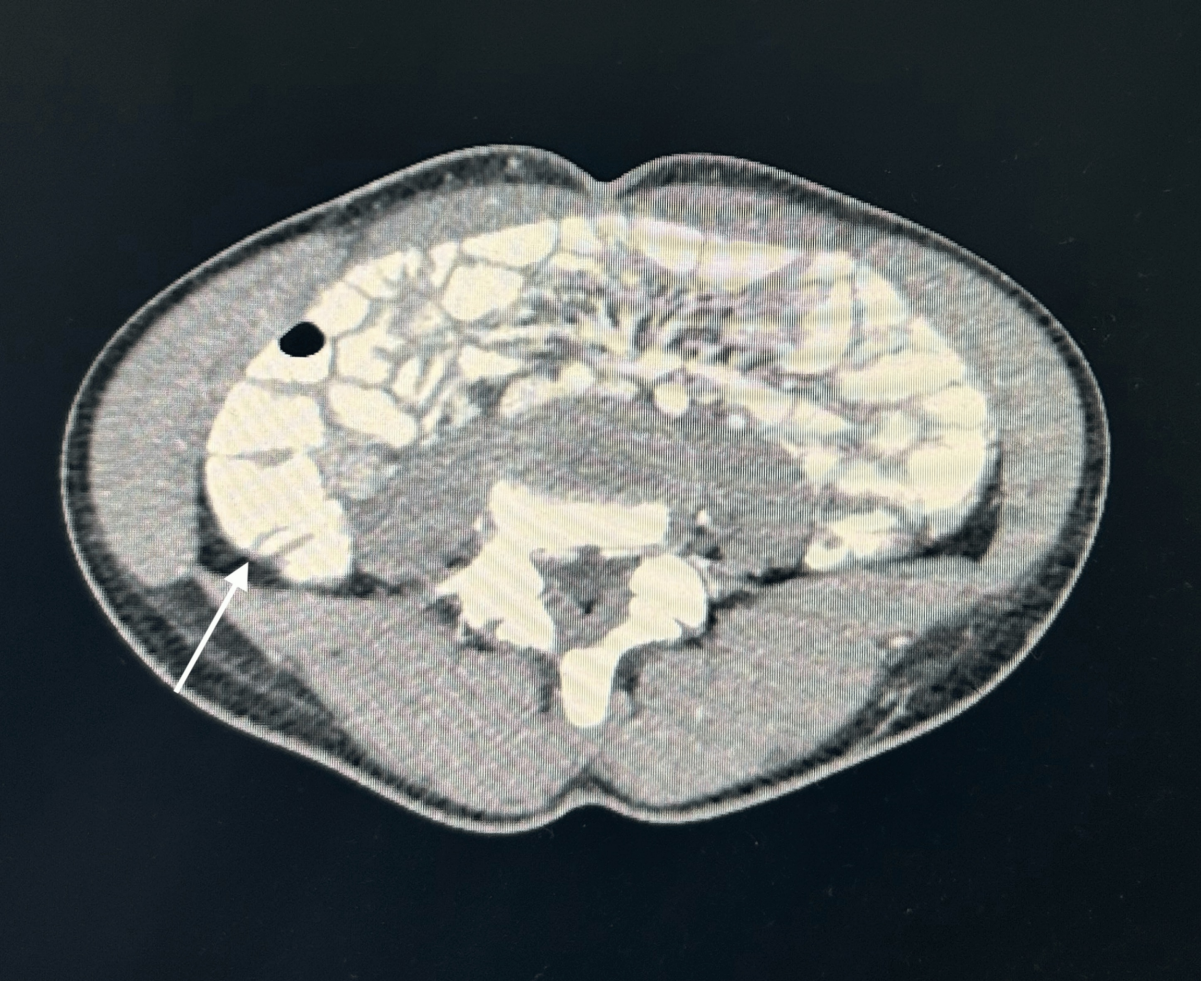
CT axial view with normal appendix CT: computed tomography

**Figure 2:**
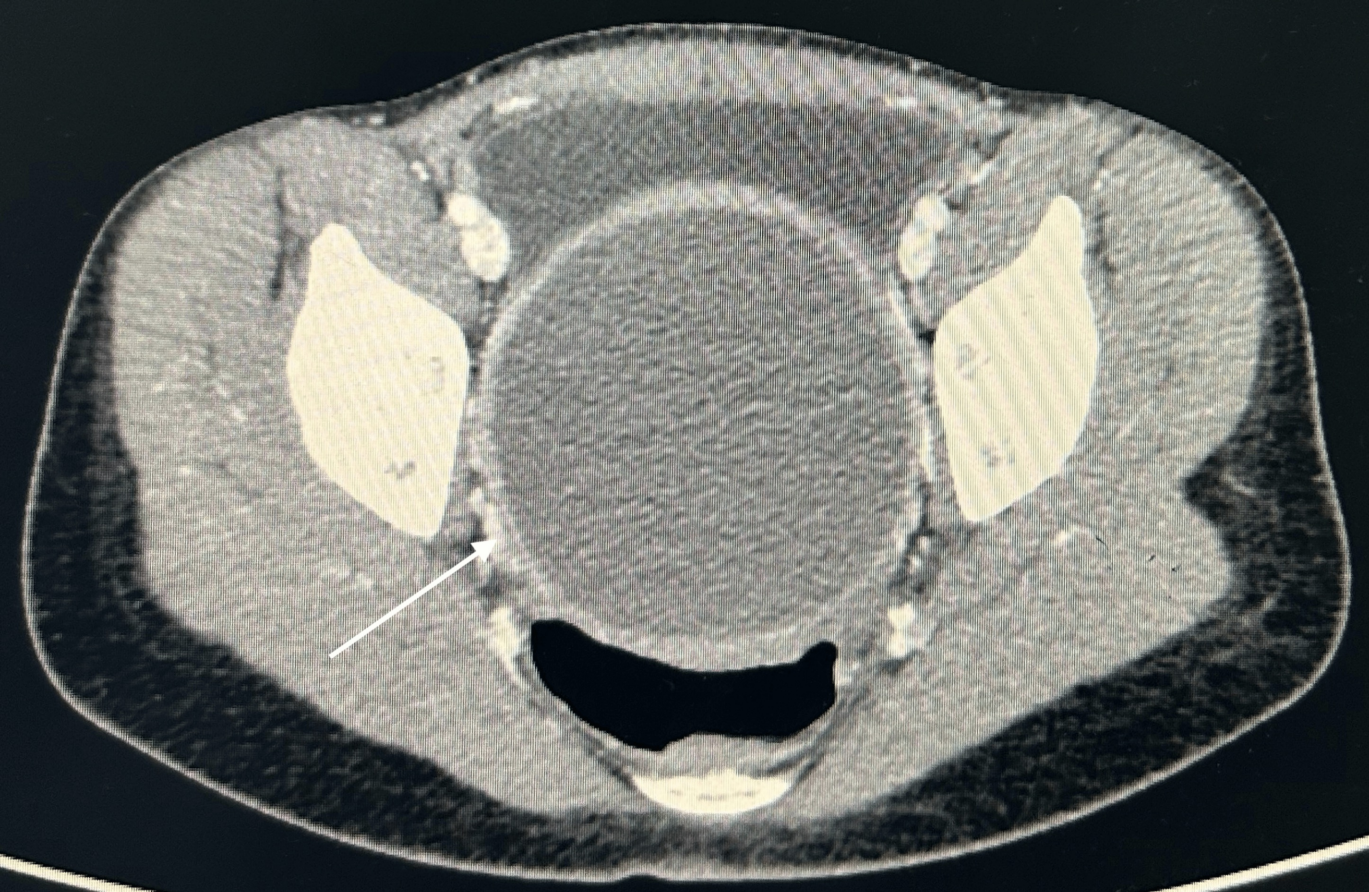
CT axial view with a fluid-filled uterine cavity CT: computed tomography

**Figure 3:**
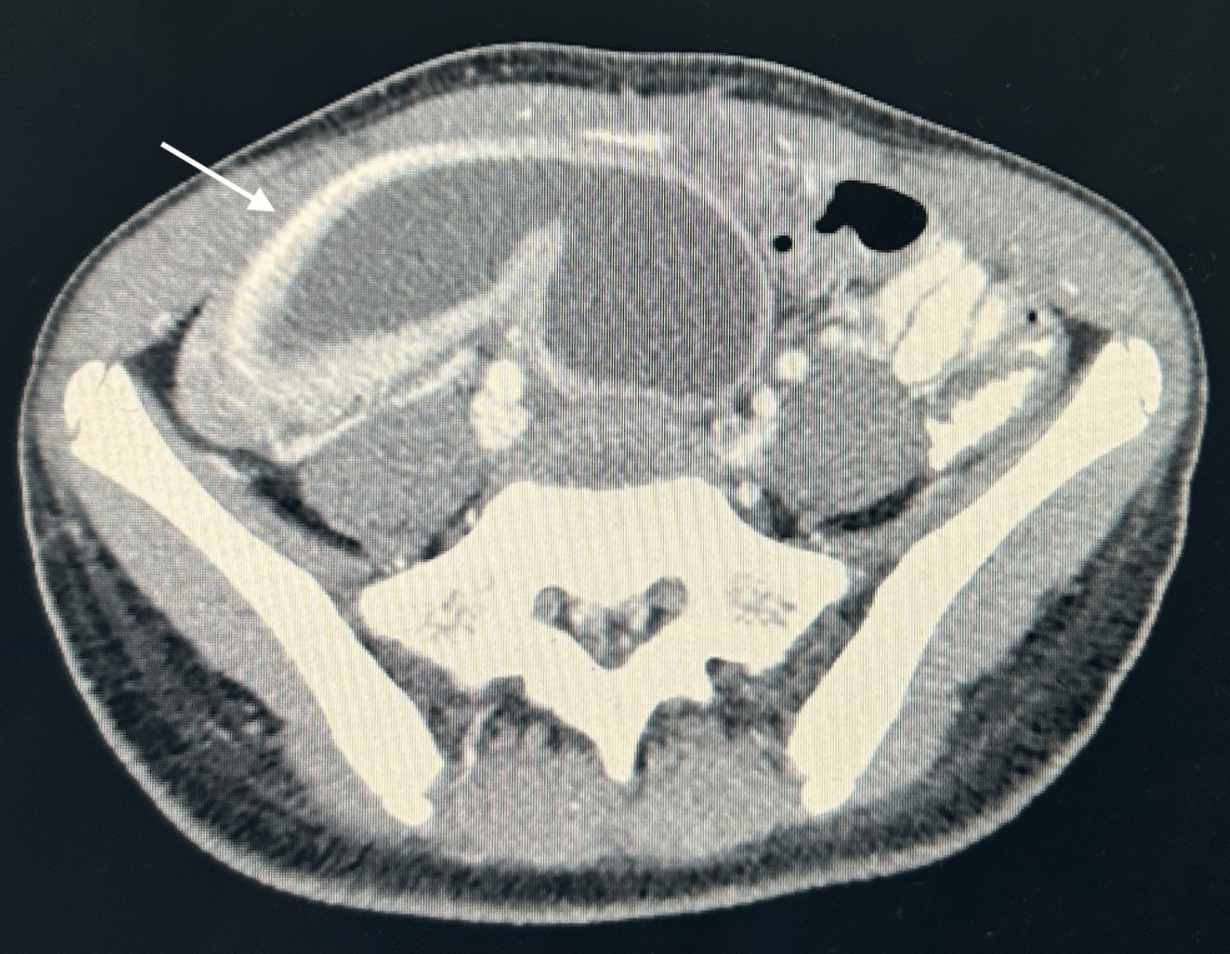
CT axial view with a fluid-filled uterine cavity CT: computed tomography

Subsequent CT imaging of the abdomen revealed massive haematometrocolpos, wherein the uterus was distended by blood and was displaced into the RIF, measuring 7.2 cm × 3.8 cm × 7.1 cm. The vagina was markedly distended at dimensions of 8.4 cm × 8.8 cm × 18.1 cm, leading to compression of the rectum and partial anterior displacement of the bladder. Both ovaries appeared normal, but were displaced (with the right ovary located in the RIF and the left positioned anteriorly and superiorly). The appendix was normal with no indications of inflammation. The bowel, liver, gallbladder, spleen, pancreas, adrenal glands, kidneys, vasculature and bony structures presented unremarkable findings. There was an absence of intra-abdominal free fluid, abnormal collections or lymphadenopathy. These results suggested haematometrocolpos, potentially attributed to an imperforate hymen, thus warranting correlation with menstrual history and gynaecological evaluation (Figures 1, 2, 3).

Following a comprehensive review of the CT report and imaging findings, the patient's case was discussed with the gynaecology department, and she was subsequently referred for further management.

## Discussion

This case highlights a 15-year-old female who presented with signs suggestive of acute appendicitis, including right iliac fossa tenderness, nausea, and normal inflammatory markers. However, instead of appendicitis, CT imaging revealed a distended uterus and a markedly blood-filled vagina, confirming the diagnosis of haematometrocolpos. Haematometrocolpos is an important differential for acute abdominal pain in young adolescent females, as, if diagnosed early, it can be treated with minimally invasive techniques and serious complications such as hydronephrosis or uterine rupture can be avoided [6]. Because these patients often present first to general surgeons rather than obstetric or gynaecological specialists, a high index of suspicion is vital, especially when the clinical picture mimics common surgical conditions like appendicitis.

In a similar case reported by Al-Buloushi et al., a young adolescent female presented to the emergency department with recurrent urinary tract infections and was initially treated and discharged. However, the persistence of these infections led the treating team to investigate further. As with our patient, her blood work was unremarkable except for mild leukocytosis. An ultrasound revealed an underlying abnormality, prompting an MRI that ultimately confirmed haematometrocolpos [7]. This case further reinforces the importance of pursuing alternative diagnoses in adolescent females with persistent or atypical presentations.

One of the challenges with haematometrocolpos is that it is often overlooked initially, leading to delayed treatment and increased risk of complications [7,8]. It is defined as a condition in which blood or secretory fluid accumulates in the vagina due to obstruction, most commonly caused by congenital anomalies, infection, or trauma [9]. The most frequent cause is an imperforate hymen, accounting for approximately 90% of all cases [6-8]. Patients typically present after puberty with symptoms such as cyclical abdominal pain, urinary retention, and amenorrhoea [2-5]. Due to overlapping clinical features, it is frequently misdiagnosed as appendicitis, urinary tract infection, pelvic inflammatory disease, or adnexal pathology, as seen in our patient [7-10]. In this case, the likely underlying cause of haematometrocolpos was an imperforate hymen. Clinically, the diagnosis may be suggested by the presence of a tense, bulging, bluish hymenal membrane on vaginal examination [9,10]. Although tumour markers like CA-125 and CA-19-9 may be elevated, they are not required for diagnosis [7].

In the emergency department, point-of-care ultrasound has become central to the evaluation of abdominal pain [8]. While not specific for haematometrocolpos, it serves as a valuable first-line tool capable of identifying Müllerian anomalies or clot accumulation within the vaginal canal [9]. MRI remains the most sensitive imaging modality, providing detailed anatomical information such as the site and integrity of obstructive anomalies. However, in this case, a CT scan was utilised, which is more readily available in acute settings where MRI access may be limited [8,9].

The pathophysiology of haematometrocolpos mimicking acute appendicitis involves more than mass effect alone. Compression of adjacent structures-including the bladder and bowel-can produce gastrointestinal and urinary symptoms [7,11]. A significant mechanism involves right-sided ureterohydronephrosis, which may explain the right iliac fossa pain observed in some cases. A distended uterus and vagina can compress the right ureter, resulting in obstruction, urinary stasis, and renal colic-like pain referred to the lower abdomen [12]. Inflammation and peritoneal irritation, potentially worsened by retrograde menstruation, may further intensify symptoms and obscure the diagnosis. Recognising these mechanisms is essential in differentiating haematometrocolpos from true acute surgical conditions [8,11].

Surgical management of haematometrocolpos depends on the underlying cause, with the primary goal of relieving the obstruction and preventing recurrence. In cases due to imperforate hymen, a hymenotomy or hymenectomy is typically performed to establish an outflow tract. These procedures are generally safe and can be completed under local or general anaesthesia [4,10]. More complex anomalies, such as a transverse vaginal septum, may require staged surgical correction to avoid complications like vaginal stenosis [13]. In our case, the patient was referred to the obstetrics and gynaecology department for definitive management.

From a general surgical perspective, recognising gynaecological causes of acute abdominal pain is critical to avoid misdiagnosis and unnecessary operative intervention. Given the overlapping clinical features between surgical and gynaecological conditions, early imaging and prompt referral are essential. Although surgical intervention is often required, medical management, such as hormonal therapy, may offer temporary symptom relief prior to surgery [6,8]. Ultimately, the choice of treatment is determined by the underlying pathology and individual patient factors, including age and comorbidities [7,8]. General surgeons should be familiar with the basic management principles and referral pathways for such presentations.

## Conclusions

Haematometrocolpos is an uncommon but important differential diagnosis in adolescent females presenting with acute abdominal pain, especially when the symptoms mimic more common conditions like appendicitis. This case underscores the necessity for a comprehensive clinical assessment, which should encompass a detailed menstrual history and a meticulous physical examination to identify any obstructive anomalies within the genital tract. Imaging, particularly ultrasound as a first-line tool, followed by CT if needed, plays a key role in confirming the diagnosis and guiding further management. Considering the overlap in clinical presentations between surgical and gynaecological pathologies, early consultation with surgical specialists and prompt imaging are imperative to avert unwarranted interventions. Although gynaecologists predominantly perform definitive treatment, the general surgeon plays a pivotal role in the early recognition, initial assessment, and exclusion of acute surgical emergencies. Early diagnosis is essential to avoid complications such as infection, retrograde menstruation, endometriosis, or delays in care. A multidisciplinary approach is essential to ensure optimal patient outcomes and reinforces the importance of considering gynaecological causes in the differential diagnosis of abdominal pain in young female patients.

## References

[1] Abdelhadi MS (2001). Acute abdominal pain in women of child-bearing age remains a diagnostic dilemma. J Family Community Med.

[2] Kim JS (2013). Acute abdominal pain in children. Pediatr Gastroenterol Hepatol Nutr.

[3] D'Agostino J (2002). Common abdominal emergencies in children. Emerg Med Clin North Am.

[4] Sokol AI, Sokol ER (2007). General Gynecology: The Requisites in Obstetrics and Gynecology. https://www.sciencedirect.com/book/9780323032476/general-gynecology.

[5] Saleh R, Katzenbach G 3rd, Espinosa J (2017). Hematometrocolpos disguised as abdominal pain. J Emerg Med.

[6] Sharma P, Shah J, Sokkary N (2024). A case of image-guided hematometrocolpos drainage requiring tissue plasminogen activator in a pediatric patient. J Surg Case Rep.

[7] Al-Buloushi N, AlBusairi S, Alenezi A, Zahir M (2023). Urinary retention complicated by hematocolpos in an adolescent girl: case report. Int J Surg Case Rep.

[8] Pearce E, Malik A (2024). Hematocolpometra diagnosed with point-of-care ultrasound in a pediatric patient with right lower quadrant abdominal pain. J Emerg Med.

[9] Tanitame K, Tanitame N, Urayama S, Ohtsu K (2021). Congenital anomalies causing hemato/hydrocolpos: imaging findings, treatments, and outcomes. Jpn J Radiol.

[10] Marino G, Alfieri N, Tessitore IV, Barba M, Manodoro S, Frigerio M (2023). Hematocolpos due to imperforate hymen: a case report and literature systematic review. Int Urogynecol J.

[11] Kotter HC, Weingrow D, Canders CP (2017). Hematometrocolpos in a pubescent girl with abdominal pain. Clin Pract Cases Emerg Med.

[12] Okur SK, Koca YS, Yıldız İ, Barut İ (2016). Right hydronephrosis as a complication of acute appendicitis. Case Rep Emerg Med.

[13] Giannakaki AI, Baroutis D, Kalampalikis A, Michala L (2025). Congenital uterine anomaly with concurrent longitudinal and transverse vaginal septa: Presentation of two cases. J Pediatr Adolesc Gynecol.

